# Association between adherence to a low carbohydrate dietary (LCD) pattern with breast milk characteristics and oxidative markers in infants’ urine: a cross-sectional study

**DOI:** 10.1186/s41043-023-00381-7

**Published:** 2023-05-06

**Authors:** Samira Karbasi, Maryam Moradi Binabaj, Zahra Khorasanchi, Milad Bideh, Asghar Zarban, Afsane Bahrami

**Affiliations:** 1 Department of Molecular Medicine, School of Medicine, Cardiovascular Diseases Research Center, Birjand University of Medical Sciences, Birjand, Iran; 2 Cellular and Molecular Research Center, Sabzevar University of Medical Sciences, Sabzevar, Iran; 3 Department of Nutrition, School of Medicine, Mashhad University of Medical Sciences, Mashhad, Iran; 4 Department of Biochemistry, Faculty of Medicine, Birjand University of Medical Sciences, Birjand, Iran; 5 Cardiovascular Diseases Research Center, Birjand University of Medical Sciences, Birjand, Iran; 6 Clinical Biochemistry Department, Faculty of Medicine, Birjand University of Medical Sciences, Birjand, Iran; 7 Clinical Research Development Unit of Akbar Hospital, Mashhad University of Medical Sciences, Mashhad, Iran; 8 Clinical Research Unit, Faculty of Medicine, Imam Reza Hospital, Mashhad University of Medical Sciences, Mashhad, Iran

**Keywords:** Breastfeeding, Oxidative stress, Low carbohydrate diet, Mother, Infant

## Abstract

**Background:**

Breast milk (BM) is a dynamic fluid that varies over time and between women. The variations in BM components are most likely associated with maternal diet quality. This study aimed to assess adherence to a low carbohydrate dietary (LCD) pattern with oxidative stress markers of BM characteristics and infants’ urine.

**Materials and methods:**

In this cross-sectional study 350 breastfeeding mothers and their infants were recruited. BM samples were collected from mothers, and urine specimens were obtained from each infant. To evaluate LCD scores, subjects were divided into 10 deciles according to the percent of energy obtained from carbohydrates, proteins, and fats. Determination of total antioxidant activity was conducted using the ferric reducing antioxidant power (FRAP), 2, 2′-diphenyl-1-picrylhydrazyl (DPPH), thiobarbituric acid reactive substances (TBARs), and Ellman’s assay. Biochemical assays of samples including calcium, total protein, and triglyceride level were also performed using commercial kits.

**Results:**

Participants with the greatest LCD pattern adherence were placed into the last quartile (Q4), and those with the minimum LCD were in the first quartile (Q1). Individuals in the highest LCD quartile had significantly higher levels of milk FRAP, thiol, and protein, as well as infant urinary FRAP and lower milk MDA levels than those in the lowest quartile. Multivariate linear regression analyses indicated that higher score of the LCD pattern was associated with a higher level of milk thiol, protein, and lower level of milk MDA (*p* < 0.05).

**Conclusion:**

Our findings show that adherence to a LCD, as defined by a low level of carbohydrates in daily food intake, is linked with improved BM quality and markers of oxidative stress in infant urine.

## Introduction

Breastfeeding is crucial for the general health of infants and is recommended by World Health Organization (WHO) as the most valuable nutrition resource for all newborn infants [1, 2]. It has been well documented that breast feeding is a good source of beneficial factors, plays an important role in infants' development, and protects them against clinical illnesses [3, 4].

Reactive oxygen species (ROS) or free radicals are chemicals with unpaired electrons when produced in excess resulting in oxidative stress (OS) [5, 6]. It is well established that OS can mediate the oxidation of different biomolecules including carbohydrates, lipids, proteins, and DNA, and ultimately induces cell and tissue damage [7]. In neonates, exposure to a milieu with a high concentration of oxygen directly after birth leads to the generation of ROS and OS induction [8]. If the imbalance between oxidative status and antioxidant system activity is sustained, finally may contribute to the pathogenesis of various diseases in newborn infants such as retinopathy, chronic lung disease, renal failure, and brain disorders [9, 10]. Studies have shown that BM provides a good antioxidant system including superoxide dismutase (SOD), glutathione peroxidase (GPx), catalase (CAT), and thiols, and it is assumed that this complex antioxidant system is associated with protective properties against oxidative damage in neonates [11, 12]. However, there is evidence demonstrating that different factors such as stage of lactation, maternal diet quality, and maternal health status could affect the BM components [13].

One of the most important sources of energy in the dietary regimen in the Asia, especially in Iran, is carbohydrates in form of rice and bread [14]. There are different methods for assessment of dietary intake patterns including a low carbohydrate diet (LCD) [15]. A LCD is a diet that is effective for weight loss by restricting dietary carbohydrates [16]. Decreasing gestational weight gain at the postpartum time points has an important role in maternal health status [17]. Carbohydrate metabolism via glucose oxidation plays a key role in enhancing OS [18], and studies have shown that a LCD diet decreases the level of OS [19, 20]. BM composition is associated with the mother's dietary pattern, and BM is a good source of different nutrition [21]. As stated above, and for the mechanisms which are associated with carbohydrates and different cellular functions, the purpose of the current study was to evaluate the association between adherence to LCD diet patterns with mothers’ BM oxidation levels during the lactation period as well as newborn infants’ urine oxidative markers.

## Materials and methods

### Study design and population

In this cross-sectional study, the study group consisted of 350 breastfeeding mothers aged between 20 and 35 years, and 350 newborn infants aged between 1 and 6 months between January and February 2021. The sample size for the study was calculated based on 80% power using the PASS ver.11 using a mean ± SD of urinary MDA (μmol TBARs/mg Cr) in the first and last tertiles of the dietary approaches to stop hypertension (DASH) diet and α = 0.05 [22]. According to this calculation and with regard to design effect (Deff) = 1.5 for cluster random sampling design, 350 participants were calculated for all 4 clusters; nevertheless, 420 participants (105 participants for each cluster) were initially recruited to account for data availability and possible exclusions and dropouts. Eligible participants were recruited from health clinics in various regions of the study city. This study was approved by the Birjand University of Medical Sciences Medical Ethics Committee, and written informed consent was obtained from participants. The exclusion criteria for this study are as follows: no previous severe medical health problems (including liver, kidney, and heart disease, immune deficiency, cancer, and anorexia nervosa) or history of drug administration in the last 6 months. A total of 20 ml of BM samples were collected from mothers, between 1 and 6 months postpartum. BM samples were obtained in the morning from primary breastfeeding. Moreover, 10 ml of first-morning urine specimens was obtained from each newborn infant by a urine bag. All samples were transferred into sterile tubes and preserved at −80  °C until the tests were performed.

### Demographic, clinical, and anthropometric data collection

A trained nurse evaluated information including the mother's age, systolic blood pressure (SBP), diastolic blood pressure (DBP), infant age, sex, and head circumference. Participants’ height and weight were measured, and body mass index (BMI) was calculated from these values as weight in kilograms divided by height in meters squared. Around the infant’s head circumference (cm) was recorded using an inelastic measuring tape to the nearest mm. Electronic scales were used to measure weight to the nearest 0.1 kg. SBP and DBP values in individuals in resting conditions were measured and repeated during the same visit.

### Nutritional evaluations

Nutritionists used face-to-face interviews to complete a 65-semi-quantitative food frequency questionnaire (FFQ) to collect the individuals' dietary data. The FFQ’s reliability and validity were previously reported in the Iranian population [23]. The average daily consumption (gram/day) for reported items was estimated using household scales after participants' information about the type, size, and frequency of consumption of each food item was gathered. For the assessment of nutritional and energy consumption, we utilized Nutritionist IV software (version 7.0; N-Squared Computing, Salem, OR) corrected for Iranian dietary items.

### Computing the LCD score

To evaluate the LCD score, subjects were divided into 10 deciles according to the percent of energy obtained from carbohydrates, proteins, and fats in their diet as previously described [24]. Briefly, participants in the lowest decile of carbohydrate intake received 9 points; those in the second decile received a score of 8, and so forth. Individuals in the highest decile received 0 points. For fats and proteins, the scoring was the same, but the order was reversed. Finally, the LCD score was defined by summing up the scores that contributed to deciles of carbohydrate, fat, and protein nutrients. Adherence to consumption of the LCD diet was defined according to the LCD score (ranging from 0 to 27). Hence, the higher score is associated with more adherence to LCD consumption [24, 25], and subjects were categorized based on the LCD score quartiles.

### Biochemical measurement assessments

To perform biochemical assays of BM samples including calcium, total protein, and triglyceride level, standard biochemical kits were used according to the manufacturer’s instructions (Pars Azmoon, Tehran, Iran). All photometric methods were conducted at 37 °C, and plates were read using an ELISA plate reader (EpochTM, BioTek, Winooski, VT, USA).

### Determination of total antioxidant status (TAS)

In general, to evaluate the TAS of mothers’ BM and newborn infants’ urine specimens, 4 different analytical methods were used which are briefly stated below.

#### Ferric reducing antioxidant power (FRAP) assay

The assay was carried out according to the method of Benzie and Strain [26]. This measurement was taken based on the reduction reaction. Ten µL of each BM or infant urine sample (1:10 diluted), standard (FeSO_4_), or blank was mixed with 250 µL of the freshly prepared working FRAP solution and incubated for 10 min at 37 °C; consequently, the final product absorption was measured at 593 nm. All samples were compared to the standard curve. All tests were carried out twice, and the results were described in µmol/L [26–28].

#### 2′-Diphenyl-1-picrylhydrazyl (DPPH) assay

A modified form of the assay introduced by Brand-Williams et al. was used [29] in the present study. This reaction is based on the reduction of DPPH radical to a steady state, and during this process, the color changes from purple to pale yellow. Finally, the remaining DPPH radical absorbance was recorded at 517 nm wavelength [30]. Briefly, 50 μl of the mother’s BM was added to 950 μl of DPPH reagent and incubated at room temperature for 10 min. Then, the resulting mixture was centrifuged at 3000 g for 3 min and changes in the absorbance intensity at 517 nm were measured against a blank of methanolic DPPH solution as a control. Each infant urine sample was centrifuged at 3000 g for 3 min and diluted 1/10, followed by adding 20 μl of each sample to 250 μl of DPPH solution. The percent (%) of DPPH scavenging activity was calculated according to the following equation:

$$\left[ {\left( {{\text{absorbance}}\;{\text{of}}\;{\text{the}}\;{\text{control}} - \frac{{{\text{absorbance}}\;{\text{of}}\;{\text{the}}\;{\text{sample}}}}{{{\text{absorbance}}\;{\text{of}}\;{\text{the}}\;{\text{control}}}}} \right)*100} \right]$$

All experiments were conducted in duplicate, and the results are expressed as µmol Trolox equivalent/L.

#### Thiobarbituric acid reactive substances (TBARs) assay

The TBARS assay was employed for the determination of lipid peroxidation content in form of malondialdehyde (MDA). This procedure is based on the thiobarbituric acid (TBA) reaction with MDA, which gives a pink pigment (TBARs) [31]. For the assay, 100 μl of each specimen (BM and urine) was added to 1 ml of TBA/trichloroacetic acid/HCl mixture. After 20 min of incubation in boiling water, the mixture was rapidly chilled. To increase the efficiency of the assay, the TBARS adducts were precipitated by adding 1 ml of *N*-butanol, and subsequently. Finally, sample fluorescence peaks were read at excitation (515 nm) and emission (553 nm) wavelengths. The measured values (μmol TBARs/L) were compared with a standard calibration curve, prepared using 1.1.2.2 tetra methoxy propane as μmol/L standard and expressed.

#### Thiol quantification (Ellman’s assay)

The free thiol group amounts in samples were measured using chromogenic Ellman’s reagent [5,5’-dithio-bis-(2-nitrobenzoic acid) (DTNB)] with slight modification [32]. Briefly, 50 µl of BM samples was added to DTNB solution (50 µl of 10 mM) and Tris/EDTA buffer (1 ml), and after the incubation period, N-butanol (650 µL) was added. The absorbance was determined at 412 nm, and the net adsorption was assessed by subtracting the apparent absorbance from the absorbance of a DTNB blank (which contained methanol instead of the samples). The results were presented as μmol/L using reducing glutathione as the standard curve [33].

### Statistical analysis

The normality assumption of the variables was assessed by the Kolmogorov–Smirnov analysis. The maternal LCD scores were categorized into quartiles. To assess continuous variables with normal distribution among quartiles, a one-way ANOVA model was applied. For the calculation of the association between the maternal LCD and different BM factors and infant urinary anti-oxidant, multivariate linear regression analysis was used. All multivariate linear regression analyses were adjusted for maternal age, BMI, energy intake, and infant age. All statistical data analyses were conducted using the statistics package IBM SPSS software (SPSS, version 16.0). The differences were determined to be statistically significant based on a *p*-value < 0.05.

## Results

The baseline demographic, anthropometric, and clinical characteristics of study groups are given in Table 1 (according to the different quartiles of the adherence to the LCD). The 350-breastfeeding mother (age between 20 and 35 years) and 350 newborn infants (age between 1 and 6 months) were classified into four groups, based on the adherence to LCD (from low to high) and obtained scores: first, Q1 (lower adherence; *n* = 79), second, Q2 (*n* = 86), third, Q3 (*n* = 72), and fourth, Q4 (higher adherence; *n* = 113). Baseline characteristics and anthropometric parameters of participants who enrolled in this study were compared for the quartiles, and the results demonstrated that the general parameters of subjects were not significantly different across LCD quartiles (Table 1), but the mother’s age was significantly lower in the lowest quartile (Q1) in comparison with the highest quartile (Q4) (*p*< 0.05).

**Table 1:** Demographic, anthropometric, and clinical characteristics of the participants according to the low carbohydrate diet (LCD) quartiles

Variables (score)	Q1 79 (22.5%)	Q4 113 (32.2%)	*p*-Values
M Age (y)	27.8 ± 5.5	29.5 ± 1.1	**0.05**
M SBP (mmHg)	102 ± 0.9	106 ± 0.9	0.16
M DBP (mmHg)	63 ± 0.5	61 ± 0.1	0.51
M BMI (Kg/m^2^)	26.4 ± 2.4	26.7 ± 3.5	0.65
I Age (days)	135.6 ± 53.9	143.6 ± 43.6	0.81
Male sex of infants (%)	22.3	27.1	0.35
I head circumference (cm)	39.8 ± 2.8	40.8 ± 1.9	0.72

Abbreviations: Dietary pattern (DP), Mother (M); Infant (I); Body mass index (BMI); Systolic blood pressure (SBP); Diastolic blood pressure (DBP)

Data presented as Mean ± SD or number (%)

A comparison of the mother’s diet food components revealed that there is a significant association between some dietary profile amounts including fruits, nuts, legumes, seeds, nuts, eggs, whole grain, red and processed meat, dairy products, refined grains, and fish with adherence to LCD. According to these data, mothers in the first quartile of LCD significantly consumed a lower amount of these dietary components in comparison with the fourth quartile (*p* < 0.05). However, our results did not indicate significant differences in vegetables and poultry consumption between the Q1 and Q4 subgroups (*p* > 0.05; Table 2).

**Table 2:** Dietary intakes of participants in different quartiles of the adherence to the LCD

Variables	LCD		
	Q1 79 (22.5%)	Q4 113 (32.2%)	*p*-Value
Dietary nutrient intake			
Total energy (kcal/d)	2157.3 ± 882	3531.6 ± 836	** < 0.001**
Carbohydrate (g/d)	270.2 ± 55.5	100.2 ± 47.2	** < 0.001**
Protein (g/d)	59.3 ± 12.2	80.6 ± 11.5	** < 0.001**
Fat (g/d)	50.6 ± 15.6	83.7 ± 10.6	** < 0.001**
Components of LCD (g/1000 kcal)			
Fruits (g/day)	266.3 ± 309	306.6 ± 360	** < 0.001**
Vegetables (g/day)	202.7 ± 187	176.6 ± 135	0.10
Nuts, legume, seed (g/day)	15.7 ± 25	17.6 ± 27	**0.03**
Whole grain (g/day)	122.0 ± 70.2	99.8 ± 61.1	** < 0.001**
Poultry (g/d)	24.3 ± 12.2	29.5 ± 12.4	0.21
Dairy products (g/d)	177.2 ± 188.1	189.9 ± 187.7	**0.006**
Red and processed meat (g/day)	29.5 ± 31.2	23.3 ± 43.3	** < 0.001**
Refined grains (g/day)	101.5 ± 31.2	150.3 ± 43.3	** < 0.001**
Fish (g/d)	12.3 ± 32.4	20.9 ± 19.5	**0.01**
Eggs (g/d)	31.5 ± 11.2	50.8 ± 13.3	**0.03**

Data presented as Mean ± SD and adjusted for energy intake

^†^Obtained from ANOVA test

Significance of bold values are *p* < 0.05

After determining total antioxidant activity in mother's milk and infant urine, we compared these data based on the LCD score. The BM of subjects in the higher quartile of adherence to LCD demonstrated a higher level of FRAP and thiol (*p* < 0.05). On the other hand, these subjects significantly showed a lower level of MDA, as an index of oxidation (*p* < 0.05). In newborn infants’ urine, FRAP (*p* < 0.05) was higher in the fourth quartile in comparison with the first quartile, and the level of MDA was higher in Q4, but it was not statistically significant. After assessment of biochemical factors, our results revealed that mothers who are more adherent to LCD dietary patterns (Q4) have higher protein levels in their BM in comparison with those in Q1 (*p* < 0.05). However, our results did not demonstrate any significant difference in the level of other biochemical factors, triglyceride, and calcium between these two groups (Table 3).

**Table 3:** BM composition and infant urinary anti-oxidant by quartiles (Q) categories of LCD

Variables	LCD		
	Q1 79 (22.5%)	Q4 113 (32.2%)	*p*-Value^a^
M DPPH (µmol Trolox equivalent /L)	311 ± 114	318 ± 110	0.71
M FRAP (µmol /L)	521 ± 140	538 ± 150	**0.02**
M MDA (μmol TBARs/L)	0.15 ± 0.11	0.12 ± 0.04	**0.04**
M Thiol (µmol /L)	75.4 ± 15.1	79.5 ± 16.9	**0.01**
M Calcium (mg/dL)	8.9 ± 1.26	9.03 ± 1.14	0.63
M Protein (g/dL)	1.17 ± 1.54	1.83 ± 1.75	**0.03**
M Triglyceride (mg/dL)	4.43 ± 2.06	4.06 ± 1.57	0.23
IU DPPH (μmol eq. trolox/L)	20.2 ± 15	20.5 ± 11	0.95
IU FRAP (µmol /L)	9.6 ± 7.6	13.8 ± 8.8	**0.041**
IU MDA (μmol TBARs/L)	1.6 ± 1.7	1.8 ± 1.1	0.19

Milk (M), Infant urine (IU), Diphenylpicrylhydrazyl (DPPH), Ferric reducing ability of plasma (FRAP), Malondialdehyde (MDA)

Q1 represents a low compliance and Q4 a high compliance with an LCD

p-value from analysis of the variance (ANOVA) for groups comparison. Significance of bold values are *p* < 0.05

Multivariate linear regression analysis indicated that a higher score for the LCD pattern was associated with a higher level of milk thiol, protein, and lower level of milk MDA. This remained also significant even after adjustment for confounders (mother age, BMI, and energy intake) (Table 4).

**Table 4:** Multivariate linear regression between score of the LCD pattern and levels of BM composition and infant urinary anti-oxidant

Variables	LCD score			
	Crude		Adjusted*	
	*β*	*P*	*β*	*P*
M DPPH	−0.86	0.12	−0.10	0.07
M FRAP	0.02	0.97	0.01	0.85
M MDA	−0.13	**0.02**	−0.12	**0.03**
M Thiol	0.10	**0.05**	0.11	**0.04**
M Calcium	−0.04	0.41	−0.06	0.30
M Protein	0.11	**0.03**	0.09	**0.05**
M Triglyceride	−0.05	0.37	−0.03	0.57
IU DPPH	0.01	0.77	0.08	0.71
IU FRAP	0.09	0.87	0.23	0.30
IU MDA	0.01	0.83	0.28	0.21

Milk (M), Infant urine (IU) Diphenylpicrylhydrazyl (DPPH), Ferric reducing ability of plasma (FRAP), Malondialdehyde (MDA)

Breast milk contents were adjusted for maternal age, maternal BMI and energy intake

Infant urine contents were adjusted for infant age

Significance of bold values are *p* < 0.05

*p < 0.0

Adjusted linear regression analysis (β, 95% confidence intervals) for content in BM and infant urinary across quartiles of LCD is shown in Table 5. Linear regression analysis demonstrated that last LCD quartile was associated with higher milk thiol (*β* = 9.25; 95% CI: 2.35–16.30), protein (*β* = 0.02; 95% CI: 0.02–0.03), and lower milk MDA levels (*β* = −0.04; 95% CI: −0.06 to −0.02 versus first LCD quartile.

**Table 5:** Adjusted linear regression analysis (β, 95% confidence intervals) for content in BM and infant urinary across quartiles of LCD

Quartiles of LCD	Milk MDA	Milk thiol	Milk protein
	β (95% CI)	β (95% CI)	β (95% CI)
Q2 vs. Q1	−0.003(−0.031 to 0.026)	−0.423 (−6.37 to 5.52)	−0.009 (−0.025 to 0.007)
Q3 vs. Q1	0.014 (−0.020 to 0.049)	10.8 (3.59 to 18.11)*	−0.006 (−0.025 to 0.014)
Q4 vs. Q1	−0.044 (−0.063 to −0.021)*	9.25 (2.35 to 16.30)*	0.025 (0.020 to 0.031)*

Malondialdehyde (MDA)

Q1 represents a low compliance and Q4 a high compliance with an LCD

*p*-Value from analysis of the variance (ANOVA) for groups comparison.

* *p* < 0.05

## Discussion

To the best of our knowledge, this is the first study that investigated the association between adherence to an LCD pattern and a mother’s BM as well as a newborn infant’s urine oxidation status. Our results present that FRAP, protein, and thiol were higher in the BM of subjects in the higher quartile of adherence to LCD. Moreover, these subjects showed a significantly lower level of MDA, as an index of oxidation. On the other hand, the level of FRAP was significantly higher in urine of infants, who their mothers were in highest adherence to LCD.

There are different types of dietary patterns, and it was well established that a mother’s diet habit and nutrition have an important role in BM composition and content, and different investigations have been conducted to identify this relationship [34, 35]. Nutritional components and antioxidant content of the mother’s milk and its quality have an important role in an infant’s development, growth, and health [36, 37]. Recently, several lines of evidence have confirmed that ROS are generated during different physiological processes, and excess ROS could have toxic effects that damage organisms, therefore antioxidative scavenging mechanisms are represented by cells (enzymatic and non-enzymatic) that protect various body tissues and living cells against OS [5]. Accumulating evidence indicated that BM contains different endogenous antioxidants [38] and milk caseins could decrease lipid peroxidation [39]. These data are in accordance with our results, which show that the level of milk FRAP and thiol as antioxidants is higher in mothers who were in highest quartile of LCD. Moreover, the level of MDA as a marker of lipid peroxidation was significantly lower in mothers in highest quartile. Also, in the present study, those who were in the highest quartile of LCD pattern consumed more fruits, nuts, legumes, seeds, refined grains, dairy products, eggs, fish, and poultry. There is evidence indicating that these nutrients exert anti-oxidant activity, and improve the antioxidant defense system in the body [40–42]. It is well documented that a high carbohydrate diet has an important role in lipotoxicity and OS [43], and consistently in our study, it has been shown that mothers who consumed more carbohydrates have more MDA and triglyceride levels in their breast milk. Caloric restriction reduces the onset of diseases related to obesity, inducing having beneficial anti-inflammatory effects, mitigating the production of free radicals, and favoring greater resistance to stress and a prolonged lifespan [44]. Modifying nutrition and regular physical activity are able to activate numerous metabolic pathways such as sirtuin 1 (SIRT1), which downregulates the production of mediators of inflammation and reduces ROS production [45].

Our results are in agreement with other results reporting that BM exerts antioxidant activity, which plays an important role in the growth and development of newborn infants and decreasing toxic effects of oxidative stress, through scavenging oxidizing agents [46–48]. It has been found that his agents could pass through the digestive system of infants [46]. In agreement with these findings, our results revealed that the level of FRAP was higher in the urine of infants who their mother were at highest quartile of LCD. Moreover, there are other studies showing that the level of antioxidant agents is higher in the urine of the infants that fed with BM [49, 50].

Consistently, it has been demonstrated that the anti-oxidant level is related to the feeding initiation after birth [51], and because this study was conducted in infants aged between 1 and 6 months, it could be assumed that maternal anti-oxidant factors deliver via BM, enter to infants body via digestive system, and subsequently increase the anti-oxidant level of newborn infants. However, different factors should be considered as agents that may contribute to the nutrient and antioxidant compositions of BM, including geographic location, history of smoking, and ethnicity [52, 53].

The amount of total energy intake in our participants in the highest quartile of LCD (3531 kcal/d) was higher than the mean energy intake reported in different investigations in developing [54] and industrialized countries [55], including Iran [56]. However, in another study conducted by Ayatollahi, the mean calorie intake of mothers during the lactation period was between 1500 and 2900 kcal/d which is lower than our results [56]. Mothers who are in the lactation period are attempting to lose weight and restrict their energy intake, and it has been shown that the LCD diet results in a decrease in energy intake, which has numerous advantages for the human body [57, 58].

Our present results indicated that mothers who are more concomitant to LCD dietary patterns have lower calcium levels in comparison with those in the lowest quartile of LCD; however, this difference was not significant. It should be noted that the maternal blood minerals including calcium level are under control and it has been proposed that the mother’s dietary content of minerals, especially calcium, has no considerable effect on the mother’s BM [59, 60].

However, the calcium level of the mother’s BM who participated in the current study [(8.9 ± 1.26 mg/dL in Q1) and (9.03 ± 1.14 mg/dL in Q4)] was lower than those in a study conducted in the UK [61] and China [62]. These differences may be due to the analytical method, genetic background, sociocultural factors, and sun exposure that may influence the vitamin D and calcium levels in breast milk [63].

The results of the current study revealed that there is no significant difference in the milk triglyceride level of mothers who are more adherent to LCD in comparison with those in low adherent to LCD. Data suggest that the major proportion of milk is a triglyceride and numerous researches have shown the important relationship between the milk’s triglyceride level and the absorbed lipids from the digestive system, which have a direct association with the dietary intake of the mother [64]. In contrast to our results, there is a study reporting that the level of fat in the BM of mothers who consumed a meal with high carbohydrates and low fat is rich in fat [65]. However, due to the direct effect of the mother's adipose tissue on the milk fat content, and the importance of energy use and body size [66], there is a need for more research to evaluate this relationship. Our data demonstrated that the level of fat intake of mothers in the more adherent to LCD is lower than those in low adherent to LCD, and consistent results are reporting that there is no association between BM fat content and energy intake in form of carbohydrates and proteins [66, 67].

It is well documented that different agents could passage from mothers’ milk to the infant’s digestive system such as hormones, drugs, and especially proteins [68]. BM includes protein storage which provides amino acids that are essential and should be available for an infant’s development and growth. Moreover, these proteins are required for biological and physiological activity in newborn infants' metabolism [69]. Based on our results, it has been shown that the level of protein obtained from dietary intake in mothers in the highest adherence to LCD, is significantly higher than those in lowest adherence to LCD. A plausible hypothesis would be that due to the passage of different nutrients from daily dietary intake of mothers to the BM, the level of protein in BM of mothers of this group is higher. Generally, important factors that affect BM content and composition within and between individuals should be considered in the breastfeeding period, which are in close association with maturity of the lactating breast [70, 71], adaptation to the infants’ changing developmental requirements [71, 72], geographical region and food supply [73, 74]. Moreover, other factors including cultural differences and lifestyle factors [75], environmental factors such as the mineral composition of the living place soil [76], and genetic backgrounds [77] are also important.

## Conclusion

Taken together, the results of the current study indicate that the LCD diet is associated with the antioxidant content of BM and infants’ urine. However, data regarding the content, characteristics, and composition of mother’s BM in different populations and regions of the world are insufficient, and there is a need for more evidence to identify the better dietary pattern for breastfeeding mothers, who want to provide their infants with adequate nourishments and important factors including anti-oxidant agents.

## Data Availability

The datasets used and/or analyzed during the current study are available from the corresponding author on reasonable request.

## Abbreviations

BM: Breast milk
FRAP: Ferric reducing antioxidant power
DPPH: 2, 2′-Diphenyl-1-picrylhydrazyl
TBARS: Thiobarbituric acid reactive substances
ROS: Reactive oxygen species
FFQ: Food frequency questionnaire
TBA: Thiobarbituric acid
MDA: Malondialdehyde
DTNB: 5,5’-Dithio-bis-(2-nitrobenzoic acid)
LCD: Low carbohydrate diet
WHO: World Health Organization
SOD: Superoxide dismutase
GPx: Glutathione peroxidase
CAT: Catalase
OS: Oxidative stress
BMI: Body mass index
TAS: Total antioxidant status
